# Suicide prevention program (SPP) in South Asian Countries: A protocol for systematic review

**DOI:** 10.12688/f1000research.132215.1

**Published:** 2023-04-20

**Authors:** Kallabi Borah, Tessy Treesa Jose, Anil Kumar Mysore Nagaraj, Lorna Moxham

**Affiliations:** 1 Psychiatric Nursing, Manipal College of Nursing, Manipal Academy of Higher Education, Manipal, Udupi, Karnataka, 576104, India; 2 Psychiatric Nursing, Manipal College of Nursing, Manipal Academy of Higher Education, Manipal, Udupi, Karnataka, 576104, India; 3 Psychiatry, Kasturba Medical College, Manipal Academy of Higher Education, Manipal, Udupi, Karnataka, 576104, India; 4 Mental Health Nursing, University of Wollongong, Wollongong, New South Wales, Australia

**Keywords:** systematic review, South Asian countries, college students, suicide prevention, suicide prevention program.

## Abstract

**Introduction:** Every year, over 703,000 individuals lose their life by suicide and many individuals attempt suicide. Suicide occurs in all age groups and is the fourth major cause of death among 15–29-year-olds globally in 2019. A suicide prevention program (SPP) is a capacity-building program that helps gatekeepers to identify the risk of suicide. The objective of the review is to determine the effectiveness of SPP on the improvement of knowledge, attitude, and gatekeeper behaviour among gatekeepers in South Asian countries.

**Methods:** The Preferred Reporting Items for Systematic Reviews and Meta-Analyses (PRISMA) and PICO (Population, Intervention, Comparison, Outcome) format will be followed in this review. This review will include all interventional studies that provided a suicide prevention program to the gatekeepers as an intervention. The full-text articles will be included from the following databases, Scopus, PubMed (MEDLINE), Cochrane, PsycINFO, Web of Science, and CINAHL, published in peer-reviewed, indexed, and English language journals from the date of inception to 2022. A grey literature search and hand-search of reference lists of the included studies will also be done. A search strategy will be developed using keywords and MeSH terms for each database. Cochrane ROB-2 tool, JBI Critical Appraisal Checklist, Critical Appraisal Skills Program (CASP), and Mixed Methods Appraisal Tool (MMAT) will be used to evaluate the quality of individual studies. Analysis of the data will be done using narrative synthesis.

**Conclusions:** This review will provide information on knowledge, attitude, and gatekeeper behaviour toward suicide prevention in college students and will be helpful for the prevention of suicide. Therefore, the authors plan to publish the review outcome through a peer-reviewed journal.

**Registration** **:** ****The review is registered in PROSPERO (CRD42023387020).

## Introduction

Suicide is an emergency problem in psychiatry. It is one of the major causes of death among today’s youth. ^1^ Several factors such as biological, psychological, and environmental factors are associated with youth suicide, varying from family issues to rapid urbanization and a weak psychological system. ^2^ Childhood abuse (physical, emotional, and sexual) also plays a role in future suicidal ideation among youth. ^3^^,^^4^

The suicide rate is increasing every year. The World Health Organization (WHO) reported that one individual dies by suicide every 40 seconds. Worldwide, every year more than 700,000 individuals take their life by suicide, and many individuals attempt suicide. ^5^ The UN’s Sustainable Development Goal (SDG) 3 is “Ensure healthy lives and promote well-being for all at all ages” and the target SDG 3.4 explains “By 2030, reduce by one-third premature mortality from non-communicable diseases through prevention and treatment and promote mental health and well-being” under which one indicator (SDG3.4.2) is the suicide mortality rate. ^6^ A total of 79% of the world’s suicide occurred in low and middle-income countries. ^7^ Worldwide, a total of 1.4% of all deaths are associated with suicide among those aged 15-24 years (Web-based Injury Statistics Query and Report System [WISQARS], 2017) and it is the fourth major cause of death among those aged 15 to 29. ^8^ The calculated age-standardized suicide rate among those aged 15 years or more was 22.0 per 100,000. ^9^

India has the highest suicide rate in the Southeast Asia region. ^10^ According to a WHO report (Sep 9, 2019), the rate of suicide in India is 16.5 per 100,000 people. ^11^ In India (2021) out of 13,089 students who died by suicide, 7,396 were male and 5,693 were female. ^12^ A review reported that compared to other high-income countries, Asia has higher average suicide rates. ^13^ Very few reviews are available on suicide in South Asia, and only India and Sri Lanka have been included in most of the reviews. ^14^

The National Crime Records Bureau (2017) reported that one student commits suicide every hour, and one of the major causes of suicide is a failure in examinations. ^15^^,^^16^ The other causes that lead to suicide among students are depression, relationship issues, psychiatric problems requiring medical attention, a history of psychiatric hospitalization, and academic obstacles. ^17^

A gatekeeper can be anyone ( *e.g.*, teachers, parents, hostel wardens, community leaders, police, layperson, counsellors, among others) who is ready to give time and effort to prevent suicide at the community level. ^18^ A gatekeeper training program is a capacity-building suicide prevention program recommended by WHO that aims to assist individuals with the skills and knowledge required to be first responders to someone who is in psychological distress and potentially suicidal and helps them to get better services as needed. As suicide is a growing problem among adolescents, suicide prevention program will help the gatekeepers identify the risk of suicide at the grass root level. Therefore, this review is intended to determine the effect of suicide prevention programs among gatekeepers on the prevention of suicide among college students in South Asia (Afghanistan, Bangladesh, Bhutan, India, Maldives, Nepal, Pakistan, and Sri Lanka) ^19^ and to improve their knowledge, attitude, and gatekeeper behaviour through suicide prevention program.

### Objective

The objective of the review is to determine the effectiveness of suicide prevention programs (SPP) on the improvement of knowledge, attitude, and gatekeeper behaviour among gatekeepers so that the number of suicide cases will be reduced among college students in South Asia countries.

## Review question

• Are suicide prevention programs effective among gatekeepers in the prevention of suicide among college students?
• What types of suicide prevention programs are effective in the prevention of suicide among college students?
• What are the components that make suicide prevention programs effective?
• Does the suicide prevention program help in the improvement of knowledge, attitude, and gatekeeper behaviour among gatekeepers?

## Methods

### Eligibility criteria

The Preferred Reporting Items for Systematic Reviews and Meta-Analyses (PRISMA) ^20^ will be used to report systematic reviews. The PICO (Population, Intervention, Comparison, Outcome) format will be adopted to define the methods of the review. The protocol was registered on PROSPERO (CRD42023387020).

*Types of studies*

All interventional studies that provide suicide prevention programs to the gatekeepers as an intervention to prevent suicide in college students, and published in indexed and peer-reviewed, English language journals from the date of inception to 2022 in South Asia will be included. A grey literature search and hand-search of reference lists of included studies will be done. The conference proceedings, reports, review papers, letters to the editor, or responses to articles, as well as studies published in other than English will not be included.

*Participants*

Gatekeepers who have undergone any suicide prevention program as an intervention will be the participants in the present review.

*Intervention*

We will include studies that provide suicide prevention programs as an intervention in the form of a workshop, different methods of teaching, and a module/booklet.

*Comparison*

We will include studies that compare the intervention group (receiving any intervention in suicide prevention) and the control group (not receiving any intervention in suicide prevention).

*Outcome measures*

The outcome measures will be suicide prevention, knowledge, attitude, and gatekeeper behaviour.

### Information sources

Primary studies will be searched by two independent authors through electronic databases: Scopus, PubMed (MEDLINE), PsycINFO, Cochrane, Web of Science, and CINAHL using MeSH terms, Emtree and synonyms of keywords of a suicide prevention program, South Asia, gatekeepers, and college students. Boolean operators (AND, OR) will be used to create specific search strategies for each database.

*Additional searches*

a) *Hand searching:* To find out additional studies, authors will hand-search reference lists of all primary studies and review articles.
b) *Grey literature:* Authors will conduct a grey literature search to find out the studies not indexed in the above-listed databases.

*Search strategy*

We include the search strategy in Figshare ( *Extended data*). ^21^

### Study records

*Data management*

RevMan 5 software ^22^ will be used for the screening and data extraction of the review. The collected search results from the databases will be kept in one folder and will be imported into EndNote and will be arranged by databases, inclusion criteria, and exclusion criteria.

*Selection process*

A stepwise approach will be followed by the authors to identify the eligibility of the studies for inclusion in this review. To identify eligibility and remove duplicates, titles and abstracts of the studies will be screened by two authors independently. The full-text article screening will be done for the potentially eligible studies. The full-text studies will be retrieved and assessed for inclusion in the review by two reviewers. A third reviewer or an independent opinion may be requested if the first two reviewers are unsure about the study’s eligibility in the analysis. The results from independent reviewers will be sent to a third reviewer, who will compare the results and compile a list of included studies. Discrepancies between the results from both reviewers will be discussed with the third reviewer until an agreement is reached. If the full-text study is not accessible through institutional membership, then the study authors will be contacted to retrieve the manuscript. The study will be included based on inclusion criteria. After eliminating the duplicate studies, a final list of included studies will be made. The reason for the excluded study and the study selection procedure will be recorded in the PRISMA flow diagram.

*Data collection process*

Following the study selection process, the extraction of the data will be completed independently by two authors. To ensure consistency in the data extraction, the authors will first pilot the data extraction tool and the extraction process on the first ten articles. Outcome data and characteristics of the study will be included if reported within the individual studies (study authors will be contacted to collect missing information relevant to this review). A data extraction form will be used to extract the data by two independent authors.

### Data items

Bibliometric information such as authors’ names, titles, journal names, publication year, and settings will be collected along with included study characteristics such as type of study, research question, objective, observation, duration, intervention, outcome variables, and key findings.

### Outcome and prioritization

The evidence generated through this review will be presented in the form of tables and figures and based on the study objectives narrative synthesis will be done.

### Risk of bias in individual studies

The Cochrane ROB-2 tool will be used to assess the risk of bias in individual studies. ^23^ Quality assessment will be performed by two authors to conclude inconsistency by consulting with a third author.

Quantitative studies

The Joanna Briggs Institute (JBI) Critical Appraisal Checklist will be used to assess the risk of bias and selection bias.

*Qualitative studies*

The Critical Appraisal Skills Program (CASP) checklist will be used to assess the quality of the studies. ^24^

*Mixed methods studies*

The risk of bias for mixed-method studies will be assessed by the Mixed Methods Appraisal Tool (MMAT). ^25^ The assessment will be done based on the following domains of bias: (i) randomization process, (ii) deviation from intervention (allocation concealment sequence), (iii) outcome assessment, (iv) incomplete outcome data, (v) selective reporting, and (vi) absolute bias. Based on the risk of bias assessment, the studies will be classified as high, or low risk of bias.

### Data synthesis

Collected data will be described and synthesized according to the type of sources, context, and key themes. The authors will perform a meta-analysis where feasible. As part of the preliminary analysis, the sensitivity and specificity of the included studies will be shown by forest plots and receiver operating characteristics curve. A summary table will be used to depict the most important aspects of the selected studies, such as the research area, and how the suicide prevention program is effective in suicide prevention. Factors gleaned from quantitative investigations will be presented in a narrative study. Thematic analysis will be done to study the qualitative data to track down the variables. The data will be coded, and subthemes will be developed after that.

*Sensitivity analysis*

To determine the low impact of quality studies on the review findings sensitivity analyses will be performed. A high or unclear risk of bias studies as identified by the ROB-2 tool will be excluded.

*Reporting bias assessment*

Reporting bias will not be assessed due to a lack of sensitive statistical methods.

### Confidence in cumulative evidence

The Grading of Recommendations Assessment, Development, and Evaluation (GRADE) guidance ^26^ will be used by two reviewers independently to assess the quality of evidence and it will be classified as high, moderate, low, or very low.

## Discussion

The gatekeeper training helps the gatekeepers to improve their knowledge and lower their judgmental attitude towards suicide prevention. ^27^ Previous studies also revealed that gatekeeper training (GKT) improves the teachers’ competency and confidence in managing suicide-risk students. ^28^^,^^29^ The study also reported that GKT brushed up the self-perceptions of college staff in working with suicidal students and improve their skill for providing intervention. ^30^

### Ethics and dissemination

Ethical clearance is not applicable as the present review will include only published articles from different databases and no human will participate in this review. A manuscript will be prepared for publication in a Scopus-indexed, peer-reviewed journal and the results will be presented at a national and international conference after the completion of the analysis.

### Strengths and limitations

The present systematic review will include interventional studies which provide suicide prevention program to the gatekeepers as an intervention. This review will focus on suicide prevention among college students. Only studies published in the English language in South Asian countries will be included.

### Study status

Formal search has not been started.

## Author contributions

Kallabi Borah: Conceptualization, analysis, methodology, supervision, validation, writing- original draft preparation, writing- review & editing.

Tessy Treesa Jose: Conceptualization, analysis, methodology, supervision, validation, visualization, writing- review & editing.

Anil Kumar Mysore Nagaraj: Conceptualization, analysis, methodology, supervision, validation, visualization, writing- review & editing.

Lorna Moxham: Conceptualization, methodology, supervision validation, visualization, writing- review & editing.

## Data Availability

No data are associated with this article. Figshare: Supplementary material 1, https://doi.org/10.6084/m9.figshare.22249906. ^31^ This project contains the following extended data:
- PRISMA flow diagram of study selection for, “Suicide prevention program (SPP) in South Asian Countries: A protocol for systematic review” Figshare: Supplementary material 3, https://doi.org/10.6084/m9.figshare.22253254. ^21^ This project contains the following extended data:
- Search strategy for “Suicide prevention program (SPP) in South Asian Countries: A protocol for the systematic review” Figshare: PRISMA-P checklist for, “Suicide prevention program (SPP) in South Asian Countries: A protocol for systematic review, https://doi.org/10.6084/m9.figshare.22252309. ^32^ Data are available under the terms of the Creative Commons Attribution 4.0 International license (CC-BY 4.0)
