# Supplementary material for: Suicide prevention program (SPP) in South Asian Countries: A protocol for systematic review
Source: F1000Res. 2023 May 30;12:425. Originally published 2023 Apr 20. [Version 2] doi: 10.12688/f1000research.132215.2 (PMC10362371; doi:10.12688/f1000research.132215.2)
Supplement: Peer review history

# Peer review: Reviewer response for version 2

**DOI:** 10.5256/f1000research.148672.r175870

**Published:** 2023-07-24

**Authors:** Stuart Leske

**Affiliations:** 1 UQ Poche Centre for Indigenous Health, The University of Queensland, Toowong, Queensland, Australia

Thank you. I have reviewed the revised further and I have no further comments to make.

Is the study design appropriate for the research question?

Yes

Is the rationale for, and objectives of, the study clearly described?

Partly

Are sufficient details of the methods provided to allow replication by others?

Partly

Are the datasets clearly presented in a useable and accessible format?

Not applicable

Reviewer Expertise:

Suicide prevention research and systematic reviews.

I confirm that I have read this submission and believe that I have an appropriate level of expertise to confirm that it is of an acceptable scientific standard.

# Peer review: Reviewer response for version 2

**DOI:** 10.5256/f1000research.148672.r182645

**Published:** 2023-07-21

**Authors:** Katerina Kavalidou

**Affiliations:** 1 National Suicide Research Foundation, University College Cork, Cork, County Cork, Ireland

Thank you for this well written protocol which will be helpful for mapping the evidence of suicide prevention programmes focused on college students in South Asian countries. It will be good if the authors can consider that suicide prevention programmes are not only helpful for preventing suicide deaths but also to support people who are experiencing suicidal thoughts and behaviours. So, beside the few minor issues that I am suggesting below, I would suggest to add the effectiveness of SPP for further outcomes, such as self-harm or suicidal ideation if possible. You could do this as secondary outcomes that you will focus on.

 Best of luck with your work and looking forward to reading your systematic review results when they will be published.                                 

**Abstract**:

 As per my general comment above - In the Introduction you state ‘…so that the number of suicide cases will be reduced among college students…’. SPP can help reduce not only suicide deaths but behaviours, so I would not restrict your introduction and overall paper objective only on suicide deaths, which are rare in comparison to the suicidal behaviours.

**Methods**:

*Outcome measures*: the authors should mention how these measures will be assessed. Are they focusing on pre-post papers only and what type of analyses are they looking for, quantitative or qualitative or both?

*Data synthesis*: ‘…and how the suicide prevention program is effective in suicide prevention’. Better to rephrase this and maybe just say ‘and the effectiveness of suicide prevention programmes’.

**Discussion**: ‘Previous literature resulted that the suicide prevention program..’, better to have ‘presented’ or ‘highlighted’ instead of resulted.

Is the study design appropriate for the research question?

Yes

Is the rationale for, and objectives of, the study clearly described?

Yes

Are sufficient details of the methods provided to allow replication by others?

Partly

Are the datasets clearly presented in a useable and accessible format?

Not applicable

Reviewer Expertise:

Suicide prevention, multimorbidity and hospital presented self-harm and suicide-related ideation.

I confirm that I have read this submission and believe that I have an appropriate level of expertise to confirm that it is of an acceptable scientific standard, however I have significant reservations, as outlined above.

# Peer review: Reviewer response for version 1

**DOI:** 10.5256/f1000research.145123.r170503

**Published:** 2023-05-10

**Authors:** Stuart Leske

**Affiliations:** 1 UQ Poche Centre for Indigenous Health, The University of Queensland, Toowong, Queensland, Australia

Thank you for the opportunity to review this systematic review protocol. The review plans to examine the effectiveness of gatekeeper training for suicide prevention in college students in South Asian countries.

 Abstract, introduction: If the 703,000 figure is taken from WHO’s suicide estimates worldwide in 2019, it’s just 703,000 (not more), or you could say ‘over 700,000’.

 Abstract, introduction: What would be the reason for calling the intervention a suicide prevention program (SPP), rather than using the term ‘gatekeeper training’? Note that all of the previous reviews in this field use the latter term:

- Yonemoto, N., Kawashima, Y., Endo, K., & Yamada, M. (2019). Implementation of gatekeeper training programs for suicide prevention in Japan: a systematic review. International journal of mental health systems, 13, 1-6.
- Morton, M., Wang, S., Tse, K., Chung, C., Bergmans, Y., Ceniti, A., ... & Rizvi, S. (2021). Gatekeeper training for friends and family of individuals at risk of suicide: a systematic review. Journal of community psychology, 49(6), 1838-1871.
- Torok, M., Calear, A. L., Smart, A., Nicolopoulos, A., & Wong, Q. (2019). Preventing adolescent suicide: A systematic review of the effectiveness and change mechanisms of suicide prevention gatekeeping training programs for teachers and parents. Journal of adolescence, 73, 100-112.
- Nasir, B. F., Hides, L., Kisely, S., Ranmuthugala, G., Nicholson, G. C., Black, E., ... & Toombs, M. (2016). The need for a culturally-tailored gatekeeper training intervention program in preventing suicide among Indigenous peoples: a systematic review. BMC psychiatry, 16, 1-7.
- Mo, P. K., Ko, T. T., & Xin, M. Q. (2018). School-based gatekeeper training programmes in enhancing gatekeepers’ cognitions and behaviours for adolescent suicide prevention: a systematic review. Child and adolescent psychiatry and mental health, 12, 1-24.
- Burnette, C., Ramchand, R., & Ayer, L. (2015). Gatekeeper training for suicide prevention: A theoretical model and review of the empirical literature. Rand health quarterly, 5(1).
- Isaac, M., Elias, B., Katz, L. Y., Belik, S. L., Deane, F. P., Enns, M. W., ... & Swampy Cree Suicide Prevention Team (12 members) 8. (2009). Gatekeeper training as a preventative intervention for suicide: a systematic review. The Canadian Journal of Psychiatry, 54(4), 260-268.
- Holmes, G., Clacy, A., Hermens, D. F., & Lagopoulos, J. (2021). The long-term efficacy of suicide prevention gatekeeper training: a systematic review. Archives of suicide research, 25(2), 177-207.
- Yonemoto, N., Kawashima, Y., Endo, K., & Yamada, M. (2019). Gatekeeper training for suicidal behaviors: A systematic review. Journal of affective disorders, 246, 506-514.

Perhaps you could say ‘gatekeeper training for suicide prevention’?

 Abstract, methods: I think you don’t need to mention PICO here, as it is within PRISMA-P.

 Abstract, methods: When mentioning ‘interventional studies’, you may need to specify study designs. Would these be any intervention study design, controlled or uncontrolled?

 Abstract, methods: If looking purely at effectiveness, the authors may not need all these study quality assessment tools. For example, the MMAT may not be needed if the authors don’t plan to synthesise qualitative data.

 Introduction, first paragraph: You could probably say that suicide is a global public health issue, as it’s typically considered to be, rather than just a problem for psychiatry.

 Introduction, first paragraph: Who does ‘today’s youth’ refer to? Is this in India or globally? Perhaps state where it ranks in the leading causes of death among these youth. The two sentences on youth may also need to be about all ages if this review does not look at gatekeeper interventions for youth specifically.

 Introduction, first paragraph: I’m not sure what is meant by a ‘weak psychological system’, but you need to revise the language here to make this term more technical.

 Introduction, second paragraph: The statement ‘The suicide rate is increasing every year’ is incorrect. WHO’s Global Health Estimates reported that ‘In the 20 years between 2000 and 2019, the global age-standardized suicide rate decreased by 36%’ - https://www.who.int/publications/i/item/9789240026643

 Introduction, second paragraph: ‘The statement that 79% of the world’s suicide occurred in low and middle-income countries.’ Is a secondary citation in the document you cite. The primary citation is WHO data from 2016, and is actually 77% rather than 79%. I recommend instead citing https://www.who.int/publications/i/item/9789240026643. The updated figure for 2019 is 77%, the same as the 2016 figure but with a better citation.

 Introduction, second paragraph: WISQARS would need to be referenced in the reference list. I understand that WISQARS is US only. You could instead cite the WHO global health estimates again, and paraphrase a statement like: ‘Suicide was the fourth leading cause of death in young people aged 15–29 years for both sexes’ https://www.who.int/publications/i/item/9789240026643. Reference 8 is a 2014 document, but is superseded now by these estimates.

 Introduction, third paragraph: I think the first sentence of this just needs updating with the WHO report again. https://www.who.int/publications/i/item/9789240026643 It now looks like Sri Lanka has a higher rate for both sexes.

 Introduction, third paragraph: I think reference 13 is outdated now, and the WHO global estimates for 2019 should be used instead. With regards to reference 14, I think you should look at the most recent review of gatekeeper training to see if studies from South Asia were included, as that is most relevant to your review and justifies why this new review is necessary.

 Introduction, fourth paragraph: Best to avoid the word ‘commits’, and say ‘dies by suicide’ instead.

 Introduction, fifth paragraph: It isn’t clear in the abstract that the review will be about college students only. Could you check that it is consistent? You also just need to cite citation 6 in the Wikipedia page, which is the Encyclopedia of Modern Asia.

 Methods, types of studies: Is it possible that the authors can include studies in languages they speak, which might include some languages other than English? This would be a major strength of the review if you could do this and found studies in your languages.

 Methods, outcome measures: I think you need to describe the outcome measures more specifically. How would you measure suicide prevention and gatekeeper behaviour? And you may just need to specify what ‘knowledge’ and ‘attitude’ refer to…

 Methods, hand searching – do you mean included studies, when you say primary studies? It might be better to say ‘included’ studies, as primary studies mean every bit of original research you find.

 Study records, data management: You just need to cite EndNote: https://support.clarivate.com/Endnote/s/article/Citing-the-EndNote-program-as-a-reference?language=en_US

 Data synthesis - What do you mean by the sensitivity and specificity of the included studies? Their ability to rule in and rule out what condition? What do you mean by ‘to track down the variables?’

 Discussion – I’m not sure the first paragraph is necessary as it’d be good to talk about your review here.

 Extended data – you don’t need to include the PRISMA flow diagram at this stage.

 Extended data – with the search terms, you just need to clarify if this is for PubMed?

 I think you might need to make the case for why this review is necessary, as I’m not sure it will find a lot of studies. You could say that previous reviews last searched the literature in February 2017 (Yonemato et al., 2019) and May 2018 (Torok et al., 2019), and you know that studies have been published since. Or maybe these reviews were limited to developed settings, although I don’t think this would be the case.

 Just related to PRISMA-P, I couldn’t see:

- Who the guarantor is for the review
- A statement on funding
- The section on outcomes and prioritization needs to discuss if any outcomes are primary and if any are secondary, and just describe the outcomes in more detail. I would see the PRISMA-P explanation and elaboration statement for an understanding of what you need to say here: http://www.bmj.com/content/349/bmj.g7647
- You would need to talk about whether you wanted to assess meta-biases with for example, a funnel plot for publication bias. I’d encourage seeing the E and E statement on this item again: http://www.bmj.com/content/349/bmj.g7647

Is the study design appropriate for the research question?

Yes

Is the rationale for, and objectives of, the study clearly described?

Partly

Are sufficient details of the methods provided to allow replication by others?

Partly

Are the datasets clearly presented in a useable and accessible format?

Not applicable

Reviewer Expertise:

Suicide prevention research and systematic reviews.

I confirm that I have read this submission and believe that I have an appropriate level of expertise to confirm that it is of an acceptable scientific standard, however I have significant reservations, as outlined above.

# Peer review: Reviewer response for version 1

**DOI:** 10.5256/f1000research.145123.r170502

**Published:** 2023-05-05

**Authors:** Sheela Pavithran

**Affiliations:** 1 Amrita College of Nursing, Amrita Institute of Medical Sciences, Kochi, Kerala, India

**Title**

 How effective is Suicide Prevention Programme (SPP) in preventing suicide among college students?

**The review**

 The authors have chosen an area of great concern to the present day - suicide among the adolescent/college students. 

 Objectives are clearly articulated in the review.   

 The study population, intervention  and the outcomes are presented clearly

 Authors have used an exhaustive search strategy and included all the major sources of evidence, a grey literature search and hand search ensured inclusion of all the relevant interventional studies that used SPP among gatekeepers to prevent suicide among the college students. This offers room for replication of the study too.

 Conceptual homogeneity could be established by the statistical methods mentioned. 

 As the study is need of the hour - the escalating incidence of suicide among the youth, a focused intervention by the key gatekeepers as appropriate is necessary.  

 Based on the proposal presented here, the systematic review can b e approved.

Is the study design appropriate for the research question?

Yes

Is the rationale for, and objectives of, the study clearly described?

Yes

Are sufficient details of the methods provided to allow replication by others?

Yes

Are the datasets clearly presented in a useable and accessible format?

Yes

Reviewer Expertise:

Chemotherapy and Breast Cancer

I confirm that I have read this submission and believe that I have an appropriate level of expertise to confirm that it is of an acceptable scientific standard.
